# Pathways of lymph node metastasis and prognosis after right hemicolectomy for cecal cancer: results from a retrospective single center

**DOI:** 10.1186/s12957-023-03148-w

**Published:** 2023-09-06

**Authors:** Liang Yu, Zhun Liu, Zhifen Chen, Xiaojie Wang, Zongbin Xu, Weizhong Jiang, Ying Huang, Huiming Lin, Pan Chi

**Affiliations:** Department of Colorectal Surgery, Fujian Medical University Union Hospital, 29 Xinquan Road, Fuzhou, 350001 Fujian Province China

**Keywords:** Cecum cancer, Right hemicolectomy, D3 lymphadenectomy, Middle colic artery

## Abstract

**Background:**

The recommended operation for cecum cancer (CC) is right hemicolectomy (RH) in some Western countries while the principle of D3 lymphadenectomy in Japan recommends resecting approximately 10 cm from the tumor edge. Therefore, the optimal surgical approach for cecum cancer (CC) remains controversial. We conducted this retrospective study to explore the pattern of lymph node metastasis and better surgical procedures for CC.

**Methods:**

A total of 224 cecum cancer patients from January 1, 2014, to December 31, 2021, were retrospectively included in the final study. The pattern of lymph node metastasis (LNM) was investigated.

**Results:**

A total of 113 (50.4%, 113/224) patients had pathologically confirmed LNM. The most frequent metastatic site was no. 201 lymph node (46%, 103/224), while 20 (8.9%, 20/224) patients had LNM in no. 202 lymph node, and 8 (3.6%, 8/224) patients had LNM in no. 203 lymph node. Only 1 (0.4%, 1/224) patient had LNM in no. 221 lymph node, four (1.8, 4/224%) patients had LNM in no. 223 lymph node, and no patients had LNM in no. 222 lymph node. LNM in no. 223 lymph node was significantly associated with a poor prognosis. Multivariate analysis indicated that LNM in no. 223 lymph node (HR = 4.59, 95% CI 1.18–17.86, *P* = 0.028) was the only independent risk factor associated with worse disease-free survival (DFS).

**Conclusions:**

The LNM in no. 223 lymph node for cecum cancer was rare. Therefore, standard right hemicolectomy excision is too extensive for most CC cases.

## Introduction

Colorectal cancer (CRC) is the third most common malignancy and the fourth leading cause of cancer-related deaths worldwide [1]. A previous study showed that up to 20% of colorectal cancer cases occurred in the cecum [2]. Currently, cancer located in the cecum, ascending colon, or proximal two-thirds of the transverse colon is classified as right-side colon cancer (RCC) [3].

**Fig. 1:**
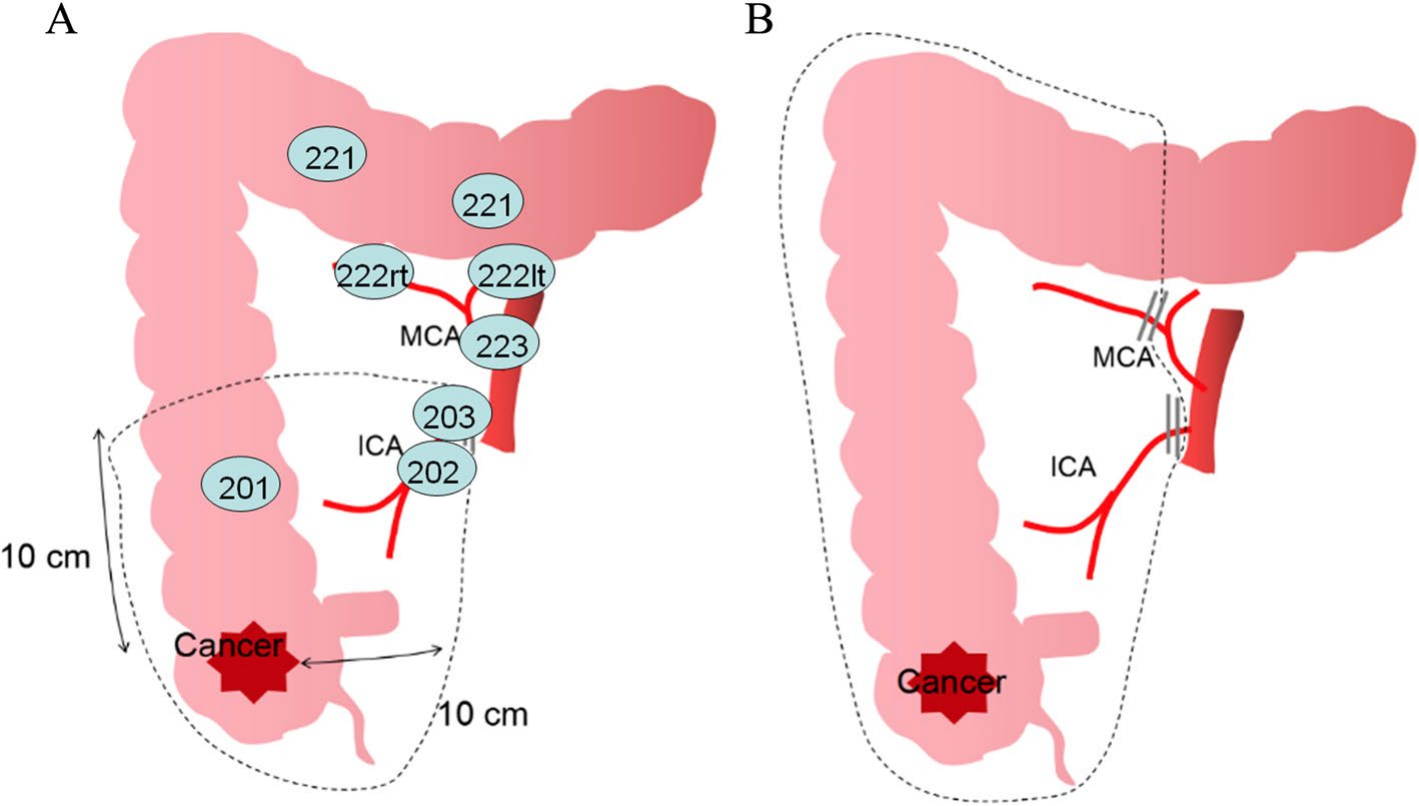
Different resection scope of Japanese D3 dissection (**A**) and complete mesocolic excision with central vascular ligation (**B**) in cecum cancer. (the figure was drawn according to the description of Kobayashi H et al [8].)

In some western countries [4, 5] and China [5], the recommended operation for cecum cancer (CC) and ascending colon cancer is right hemicolectomy (RH) with the principle of complete mesocolic excision (CME) with central vascular ligation (CVL). The range of bowel resection of RH is from the terminal ileum to the proximal transverse colon, with the high ligation of the ileocolic artery (ICA), the right colic artery (RCA), if present, and the right branch of the middle colic vessel and removal of the lymph nodes (LN), including the pericolic, ileocolic, right colic, and right branch of the middle colic [4]. However, in Japan, according to the principle of D3 lymphadenectomy defined by the Japanese Society for Cancer of Colon and Rectum (JSCCR), only the LN around the ICA should be dissected for CC if the RCA is not-existent, and the colon should be resected approximately 10 cm from the tumor edge [6]. For CC, the ICA is the primary feeding artery, and it is sufficient to dissect the regional LN within the scope of the mesocolon 10 cm from the tumor [7]. Therefore, the resection area of the mesentery and lymph dissection in the CME principle is significantly larger than that in the D3 principle for CC [8] (Fig. 1).

Interestingly, in the 8th edition Cancer Staging Manual of American Joint Committee on Cancer (AJCC), the regional LNs for cecum cancer only include LNs distributed in the pericolic, ileocolic artery, and right colic artery [9], which indicates that for localized CC, there is no need to dissect lymph nodes around the middle colic artery (MCA) and ligate the MCA during the surgical procedure.

To find a better extent of LN dissection for CC, we conducted this retrospective study to explore the distribution feature of metastatic LN for CC and the oncologic outcomes of CC.

## Materials and methods

### Patients

This was a retrospective study conducted at the Fujian Medical University Union Hospital (FMUUH). Patients who were diagnosed with cecum cancer underwent primary resection from January 1, 2014 to December 31, 2021 were reviewed. The inclusion criteria of our study were as follows: (1) pathologically proven cecal adenocarcinoma; (2) without distant metastatic disease; and (3) received RH without neoadjuvant therapy. The exclusion criteria were as follows: (1) metachronous colorectal cancer; (2) familial adenomatous polyposis (FAP); (3) multiple primary tumors; (4) palliative surgery; and (5) emergent operation. Patients met the criteria and were included in the final study. The baseline clinicopathological characteristics were collected from the patients’ medical records.

This retrospective study was approved by the institutional research ethics committee of FMUUH (2022KY095), and the study was conducted in accordance with the Helsinki Declaration (64th WMA General Assembly, Fortaleza, Brazil, October 2013).

### Surgical information

**Fig. 2:**
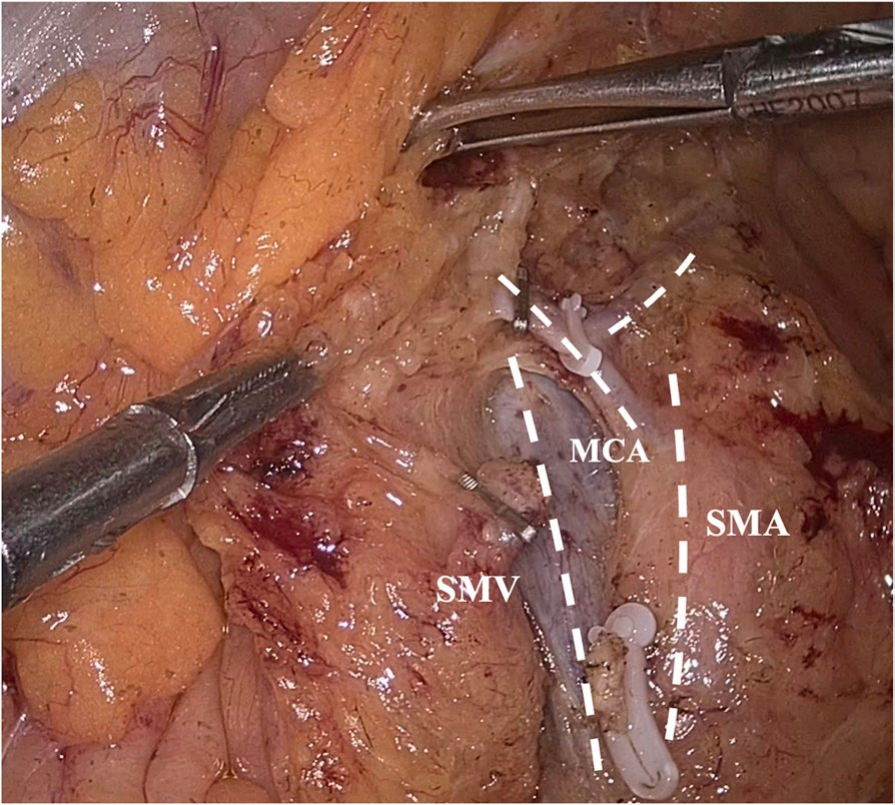
The right branch of middle colic artery was ligated with the left branch of middle colic artery preserved in right hemicolectomy

All patients underwent the RH procedure with the principle of CME with CVL by laparoscopic or open surgery, and the operations were performed by experienced senior surgeon of colorectal cancer. Usually, the surface of the superior mesenteric vein (SMV) was exposed, and the main LN around the root of the ileocolic artery, the right colic artery (if present), and the middle colic artery were dissected. The right branch of the MCA was ligated with the left branch of the MCA preserved (Fig. 2). The lymph nodes along the gastroepiploic vasculature were not dissected routinely for cecum cancer in our institution.

After the specimen was removed from the body, a colorectal surgeon classified lymph nodes according to their positions in the resected specimens, and each sample was assessed histologically. The stations of the LNs were classified according to the JSCCR [10] as follows: Pericolic LNs, which were defined as along the marginal arteries and near the bowel wall, including the region fed by the ileocolic artery (201), the right colic artery (211), and the middle colic artery (221); Intermediate LNs, which included the nodes along the ileocolic artery (202), nodes along the right colic artery (212), and the nodes along the middle colic artery (222); and the Main LNs, which were defined as that at the origin of the arteries along the superior mesenteric artery (SMA), including ileocolic root nodes (203), right colic root nodes (213), and middle colic root nodes (223). For patients without RCA, no. 211, no. 212, and no. 213 lymph nodes were absent. All samples dissected from the surgical specimens were sent for pathological examination.

### Outcome measures

The outcomes measured were operative time, the number of LN dissections, the rate of LN metastasis, and the incidence of LN metastasis according to its positions (no. 201, no. 202, no. 203…). Long-term results investigated were disease-free survival (RFS) rate and overall survival (OS) rate. Recurrence was defined as a radiologically or pathologically proven local or systemic metastasis, while OS was defined as death by any cause reported during follow-up.

### Statistical analysis

Categorical variables were presented as frequencies and percentages (%), and continuous variables were described as the mean with standard deviation (mean ± SD) or median. The Kaplan‒Meier method and the log-rank test were used for survival analysis. Multivariate analysis was conducted using a Cox proportional hazards model. All analyses were performed with SPSS software program, version 23.0 for MAC (SPSS Inc., Chicago, IL, USA). A two-tailed *P* value < 0.05 was considered statistically significant.

## Results

### Study population

**Fig. 3:**
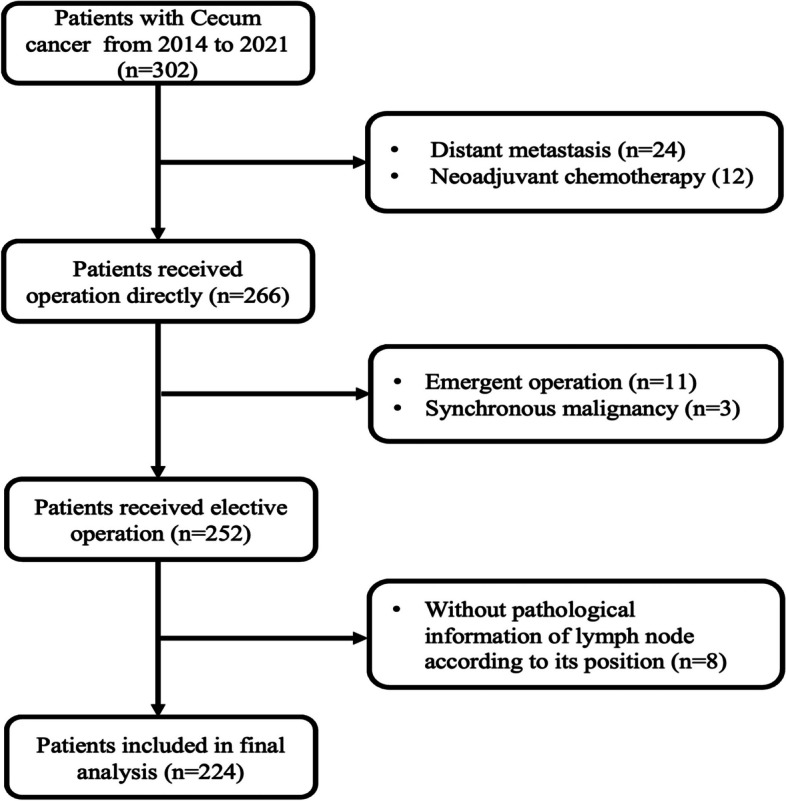
Patient selection

There were 302 patients who underwent the RH procedure for cecal carcinoma from January 1, 2014 to December 31, 2021 in our hospital, and 224 patients were included in the final analysis (Fig. 3). The mean age of 224 patients was 62.5 (26–91), and 54.5% were male. There were 15 (6.7%) patients receiving open surgery and 209 (93.3%) patients receiving laparoscopic surgery. The mean time of the operation was 146.7 (112–266) min, and the mean number of LN dissections was 35.9 (8–80)(Table 1). There were 105 (46.9%) patients with a right colic artery (RCA) and 119 (53.1%) patients without an RCA.

**Table 1:** Clinicopathological characteristics of patients

Characteristic	Value (%)
Mean age (year ± SD) (range)	62.5 ± 12.4 (26–91)
Gender, *n*(%)	
Male	122 (54.5)
Female	102 (45.5)
CEA	
> 5 ng/ml	95 (42.4)
≤ 5 ng/ml	129 (57.6)
CA19-9	
> 37 U/ml	55 (24.6)
≤ 37 U/ml	169 (76.4)
Hospital stays after surgery (mean ± SD) (range)	8.7 ± 5.2 (4–44)
Lymph nodes harvested (mean ± SD) (range)	35.9 ± (3.7) (8–80)
Histological differentiation, *n *(%)	
Well differentiated	16 (7.1)
Moderately differentiated	158 (70.5)
Poor differentiated	10 (4.5)
Mucinous	40 (17.9)
pT category, *n* (%)	
T1	5 (2.2)
T2	17 (7.6)
T3	160 (71.4)
T4a	23 (10.3)
T4b	19 (8.5)
pN category, *n* (%)	
N0	111 (49.6)
N1	74 (33.0)
N2	39 (17.4)
pTNM stage, *n* (%)	
I	19 (8.5)
II	92 (41.1)
III	113 (50.4)
Vascular invasion, *n* (%)	
Yes	70 (31.3)
No	154(68.7)
Perineural invasion	
Yes	60 (26.8)
No	164(73.2)

### Lymph node metastasis pattern

**Fig. 4:**
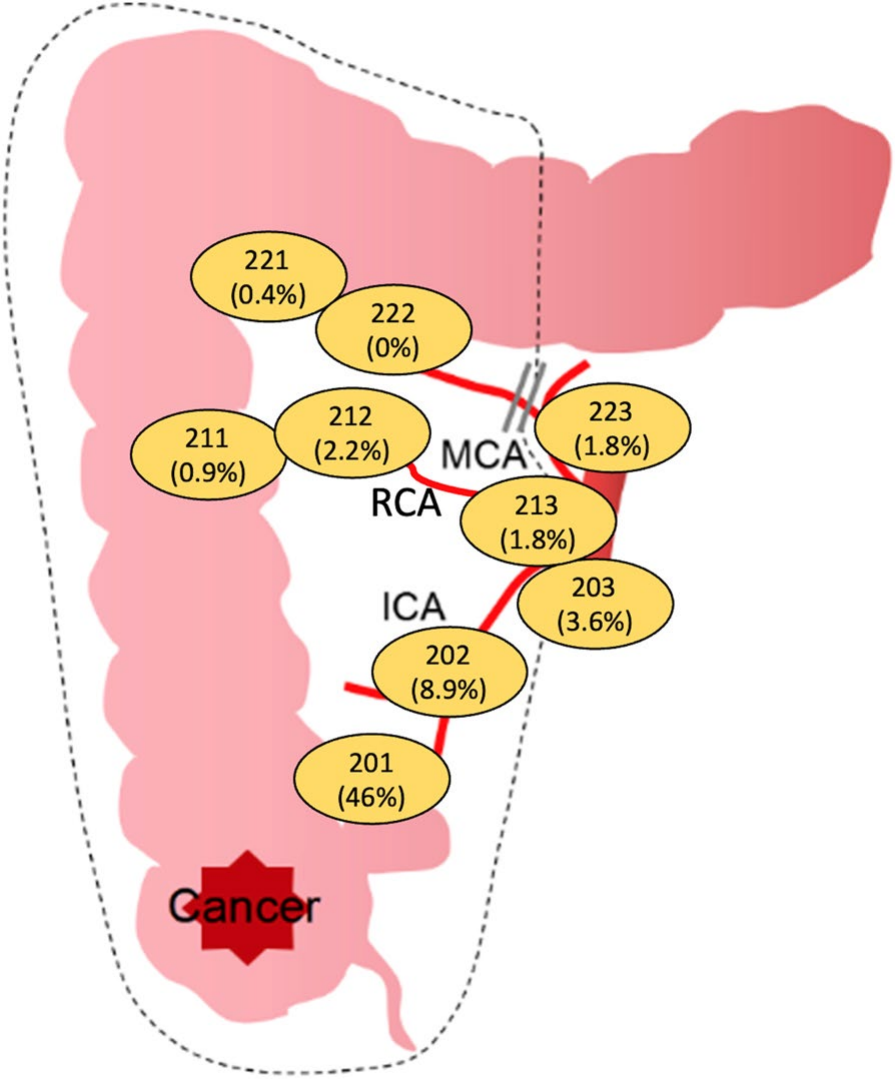
Location of lymph node metastases in patients with cecal colon cancer

In total, 113 (50.4%, 113/224) patients had pathologically confirmed lymph node metastasis, and the mean number of metastatic LNs was 4.5 ± 5.8 (range from 1 to 43). The most frequent metastatic site was no. 201 lymph node (46%, 103/224), while 20 (8.9%, 20/224) patients had metastases in No.202 lymph node, and 8 (3.6%, 8/224) patients had positive LNs in no. 203 lymph node (Fig. 4). There were 52 patients with both RCA and LN metastasis; among these patients, the metastasis rates in no. 211, no. 212, and no. 213 lymph node were 2 (0.9%, 2/224), 5 (2.2%, 5/224), and 4 (1.8%, 4/224), respectively. Only one (0.4%, 1/224) patient had lymph node metastasis in no. 221, four (1.8%, 4/224) patients had LN metastasis in no. 223, and no patients had lymph node metastasis in no. 222 (Fig. 4). Patients who had no. 223 lymph node metastases all had more than 10 LN metastases and some factors indicative of a poor prognosis (poor differentiation, vascular invasion, or perineural invasion) and no patients had no. 223 lymph node metastases alone (Table 2).

**Table 2:** Clinicopathological characteristics of patients who had lymph node metastasis in no. 223

Patient	Sex	Year	CEA (ng/L)	CA199 (U/ml)	Operative approach	Operative time (min)	Had RCA	Number of LN harvested	Number of LNM	Histological grade	Vascular invasion	Perineural invasion	Number of LNM in no. 223	Preoperative radiologic findings of LNM in no. 223
A	M	69	1.7	16.1	Laparoscopic	135	No	52	11	moderate	Yes	Yes	3	No
B	M	64	6.7	49.0	Laparoscopic	142	Yes	33	13	low	No	Yes	4	No
C	F	53	11.2	29.2	Laparoscopic	130	No	32	13	low	Yes	Yes	1	Yes
D	F	44	15.8	0.67	Laparoscopic	140	Yes	53	43	moderate	Yes	No	11	Yes

*LN* lymph node, *LNM* lymph node metastasis, *RCA* right colic artery

When analyzing the preoperative radiologic examination of four patients who had LN metastasis in no. 223, only two of four patients had enlarged LNs in no. 223, and another two patients were considered to have no LN metastasis (cTxN0) by preoperative radiologic examination.

### Survival analysis

**Fig. 5:**
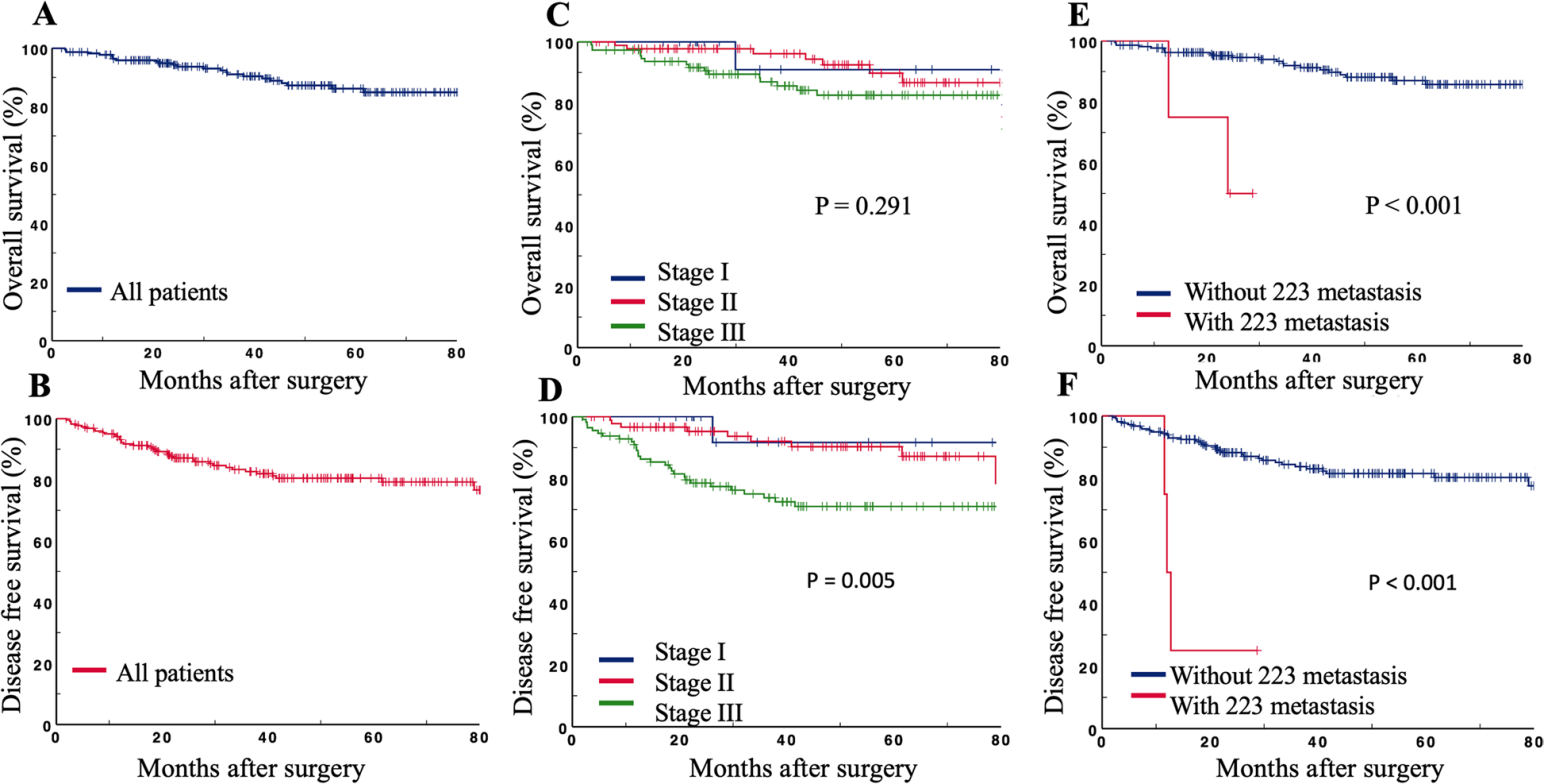
Overall survival rate (**A**) and disease-free survival rate (**B**) for 224 cecum cancer patients; overall survival rate (**C**) and disease-free survival rate (**D**) for patients with different TNM stage; patients with lymph nodes metastasis in no. 223 had significantly worse overall survival rate (**E**) and disease-free survival rate (**F**) than that without lymph node metastasis in no. 223

The median follow-up time was 45.4 months (1.9–101.0 months), with a 5-year overall survival (OS) of 86.2% and a 5-year disease-free survival (DFS) rate of 80.5% for all 224 patients (Fig. 5A, B). The 5-year OS rates were 90.9%, 89.8%, and 82.6% for patients with stage I, stage II, and stage III disease, respectively (Fig. 5C). The 5-year DFS rates were 91.7%, 90.3%, and 71.0% for patients with stage I, stage II, and stage III disease, respectively (Fig. 5D). For patients who had lymph node metastasis, the no. 223 lymph node metastases were a significant factor for a poor prognosis (Fig. 5E, F).

Univariate analysis indicated that pTNM stage (*P* = 0.004), the number of negative LNs (HR = 0.97, 95% CI 0.95–0.99, *P* = 0.025), vascular invasion (HR = 2.40, 95% CI 1.28–4.52, *P* = 0.006), adjuvant chemotherapy (HR = 0.48, 95% CI 0.25–0.93, *P* = 0.025), lymph node metastasis in no. 201 (HR = 2.48, 95% CI 1.28–4.83, *P* = 0.006), lymph node metastasis in no. 202 (HR = 3.19, 95% CI 1.46–6.95, *P* = 0.004), and lymph node metastasis in no. 223 (HR = 9.13, 95% CI 2.73–30.5, *P* < 0.001) were associated with DFS (Table 3). According to the results of the multivariate analysis, lymph node metastasis in no. 223 (HR = 4.59, 95% CI 1.18–17.86, *P* = 0.028) was the only independent risk factor associated with DFS after multivariate analysis (Table 3).

**Table 3:** Univariate and multivariate analysis of prognostic factor of disease-free survival

Characteristics	Univariate analysis			Multivariate analysis		
	HR	95%CI	*P* value	HR	95%CI	*P* value
Age	0.99	0.97–1.02	0.701			
Gender			0.534			
Male	Reference					
Female	0.82	0.43–1.55				
pT stage			0.294			
T1–2	Reference					
T3–4	2.14	0.52–8.89				
pN stage			0.051			
N0	Reference					
N +	1.89	0.99–3.62				
pTNM stage			**0.004**			0.393
I	Reference		0.009	Reference		
II	1.92	0.24–15.18	0.536	1.76	0.22–13.96	0.593
III	5.42	0.74–39.78	0.097	4.69	0.46–48.06	0.192
CEA			0.571			
< = 5 ng/L	Reference					
> 5 ng/L	1.20	0.64–2.25				
CA199			0.947			
< = 37 U/ml	Reference					
> 37 U/ml	1.02	0.49–2.10				
Number of lymph nodes	0.99	0.97–1.02	0.600			
Number of negative lymph nodes	0.97	0.95–0.99	**0.025**			
Perineural invasion			0.057			
No	Reference					
Yes	1.88	0.98–3.59				
Vascular invasion			**0.006**			0.116
No	Reference			Reference		
Yes	2.40	1.28–4.52		0.59	0.30–1.14	
Histological differentiation			0.559			
Well differentiated	Reference					
Moderately differentiated	0.34	0.04–2.71	0.307			
Poor differentiated	0.94	0.43–2.07	0.875			
Mucinous	1.48	0.39–5.58	0.566			
Adjuvant chemotherapy			**0.025**			
No	Reference					
Yes	0.48	0.25–0.93				
Metastasis in no. 201 LNs			**0.006**			0.673
No	Reference			Reference		
Yes	2.48	1.28–4.83		1.31	0.38–4.52	
Metastasis in no. 202 LNs			**0.004**			0.350
No	Reference			Reference		
Yes	3.19	1.46–6.95		1.54	0.62–3.84	
Metastasis in no. 203 LNs			0.073			
No	Reference					
Yes	2.94	0.91–9.57				
Metastasis in no. 223 LNs			** < 0.001**			**0.028**
No	Reference			Reference		
Yes	9.13	2.73–30.5		4.59	1.18–17.86	

*P* value were calculated using the log-rank test for univariate analysis and COX proportional hazards model for multivariate analysis. Only variables that *P* < 0.001 were included in multivariate analysis to make results more solid

*LNs* lymph nodes

## Discussion

Carcinomas of the cecum and ascending colon are both defined as RCC and are always recommended to receive the same operation procedure, usually right hemicolectomy, in previous clinical trials and textbooks [4]. Generally, the standard RH requires a high vascular tie of the right branch of the MCA [4, 11]. However, the LNs around the MCA do not belong to the regional LNs according to the 8th edition Cancer Staging Manual of AJCC [9]. Therefore, standard RH for CC patients may result in excessive excision of the distal colon and lymphadenectomy.

A retrospective study that included 2084 cancer patients of the cecal and ascending colon showed no benefit from more extended mesenteric resection, indicating that there is no need to extend the mesenteric resection to involve the middle colic vessels in cancer of the cecum or ascending colon [12]. Another systematic review of 17 studies indicated that compared with traditional right hemicolectomy, CME did not demonstrate oncological superiority in terms of survival with the data available, but it had not been proven inferior to traditional surgery in terms of feasibility and safety [13]. In contrast, Gennero et al. reported that CME significantly improved the long-term oncological impact on right-sided colon cancer and did not increase the risk of postoperative complications [14]. Therefore, whether the CME + CVL procedure is suitable for right-colon cancer is inconclusive. In fact, the RH procedure with CME + CVL might be suitable for ascending or hepatic flexure colon cancer but not for CC because the resection scope of standard RH is excessive for CC, and there is not enough evidence to support this opinion because no studies have evaluated the best surgical scope and procedure for CC.

In our study, LN metastasis was detected in only 1.8% of cecum cancer patients in no. 223, and one of them had LN metastasis in the no. 221. Park et al. reported 6.1% of patients had lymph node metastasis along the right branch of the MCA in cecum cancer; however, the study only included a small number of patients with cecum cancer (75 patients), and it did not explain how that number was calculated [15]. In the current study, every patient who had lymph node metastasis in no. 223 had more than 10 metastatic LNs in total, which were distributed in different mesentery regions, and no patients only had LN metastasis in no. 223. These patients had significantly worse survival than patients without LN metastasis in no. 223. These results indicated that metastasis to no. 223 LNs is a rare event and usually occurred in the advanced period of the disease in patients with cecum cancer. What is even more concerning, in the multivariate analysis in terms of DFS, LN metastasis in the no. 223 was the only factor that was significantly associated with DFS. Thus, metastasis in no. 223 indicates a poor prognosis, and whether the dissection of LNs in this area could improve the prognosis is unknown.

Although many studies found that the CME + CVL procedure did not increase the perioperative complications in right-sided colon cancer compared with “conventional” colectomy [14, 16, 17], there were also some studies indicating that CME + CVL was associated with more intraoperative organ injuries and nonsurgical complications than ‘conventional’ resection [18, 19]. Compared to Japanese D3 dissection for CC, RH with CME + CVL should dissect the Henle trunk and MCA, which makes the operation more complicated and more likely to result in intraoperative complications, including bleeding or injury to the duodenum. Olofsson et al. evaluated the short- and long-term outcomes in three variations of RH based on the position of the vascular ligature in the mesentery and found that it was not necessary to extend the mesenteric resection to involve the MCA in cancer of the cecum or ascending colon because no benefit was found by a greater extent of mesenteric resection. In contrast, increasing perioperative mortality by extensive mesenteric resection was noted [12]. Our data demonstrated that Japanese D3 dissection, whose resection scope is smaller than standard RH with the principle of CME + CVL, is sufficient for most cecum cancer patients because metastasis to the no. 223 is rare, and dissection of no. 223 LNs can be performed in relatively advanced tumors because metastasis to no. 223 occurs almost exclusively in locally advanced patients. There is no need to perform prophylactic dissection of the no. 223 in cecum cancer patients who have no evidence of metastasis in the no. 223. Of course, a randomized controlled study is needed to explore the best scope of the operation for cecum cancer.

There were some limitations to our current study. Firstly, the study was subject to information and selection biases because this was a single-institution, retrospective study. Secondly, the peri-operative and oncological results were not compared between the CME + CVL group and the D3 group because CME + CVL is a routine procedure for right-side colon cancer in our department. We are comparing the two surgical procedures in different medical centers in China and Japan in our future studies. Finally, the sample of patients with LN metastasis was too small to perform a proper oncological comparison, and the follow-up period was short (range from 12.7 to 28.7 months), so the prognosis analysis exhibited bias.

In conclusion, the incidence of no. 223 LN metastasis in cecum cancer is very rare. The standard right hemicolectomy which involve resection of no. 223 for CC patients may result in excessive excision of the distal colon and lymphadenectomy. Further study is needed to explore the best surgical strategy for CC.

## Data Availability

The datasets used and/or analyzed during the current study are available from the corresponding author on reasonable request.

## Abbreviations

CC: Cecum cancer
LNM: Lymph node metastasis
CRC: Colorectal cancer
RCC: Right-side colon cancer
RH: Right hemicolectomy
CME: Complete mesocolic excision
CVL: Central vascular ligation
ICA: Ileocolic artery
RCA: Right colic artery
JSCCR: Japanese Society for Cancer of Colon and Rectum
AJCC: American Joint Committee on Cancer
MCA: Middle colic artery
SMV: Superior mesenteric vein
OS: Overall survival
DFS: Disease-free survival
